# An experimental study of phage mediated bactericidal selection & emergence of the El Tor *Vibrio cholerae*

**Published:** 2011-02

**Authors:** M. Shamim Hasan Zahid, Zaved Waise, M. Kamruzzaman, A.N. Ghosh, G. Balakrish Nair, S.A.M. Khairul Bashar, John J. Mekalanos, Shah M. Faruque

**Affiliations:** * *Molecular Genetics Laboratory, International Centre for Diarrhoeal Disease Research, Bangladesh, Dhaka, Bangladesh*; ** *National Institute of Cholera & Enteric Diseases, Kolkata, India*; + *Department of Life Sciences, North South University, Dhaka, Bangladesh*; # *Department of Microbiology & Molecular Genetics, Harvard Medical School, Boston, Massachusetts, USA*

**Keywords:** Classical biotype, El Tor biotype, toxigenic *Vibrio cholerae*, vibriophage

## Abstract

**Background & objectives:**

Factor causing the elimination of the classical biotype of *Vibrio cholerae* O1, and its replacement by the El Tor biotype causing the 7^th^ cholera pandemic are unclear. Possible ability of the El Tor strains to adapt better than the classical strains to undefined environmental forces have been largely implicated for the change. Here we describe an environmental bacteriophage designated JSF9 which might have contributed to the range of factors.

**Methods:**

Competition assays were conducted in the infant mice model and in microcosms between representative El Tor and classical biotype strains in the absence or in the presence of JSF9 phage.

**Results:**

The JSF9 phage was found to kill classical strains and favour enrichment of El Tor strains, when mixtures containing strains of the two biotypes and JSF9 phage were subjected to alternate passage in infant mice and in samples of environmental water. Spontaneous derivatives of the classical biotype strains, as well as transposon mutants which developed resistance to JSF9 phage were found to be defective in colonization in the infant mouse model.

**Interpretation & conclusions:**

These results suggest that in addition to other factors, the inherent ability of El Tor biotype strains to evade predation by JSF9 or similar phages which kill classical biotype strains, might have enhanced the emergence of El Tor strains as the predominant pandemic biotype.

Bacteriophages contribute to the evolution of bacteria by mediating horizontal gene transfer and genomic rearrangements, as well as by bactericidal selection, in which bacterial strains that are able to resist phage predation have an advantage over the susceptible strains in their competition for survival^1^–^3^. Toxigenic *Vibrio cholerae*, the causative agent of the epidemic diarrhoeal disease cholera, interacts with diverse phages, which can promote genetic diversity and/or cause selective enrichment of particular bacterial clones^2^.

Historically, cholera is an ancient disease with the occurrence of seven distinct pandemics since the first pandemic of cholera recorded in 1817, but the disease still affects millions of people. *V. cholerae* O1 belongs to two biotypes, namely classical and El Tor which differ in certain phenotypic and genetic characteristics^4^,^5^. The current 7^th^ pandemic of cholera which originated in Indonesia in 1961 is the most extensive in geographic spread and duration, and the causative agent is *V. cholerae* O1 of the El Tor biotype. The classical biotype, which caused the 6^th^ pandemic, is now presumably extinct. After a period during the 1970-1980 when both classical and El Tor strains co-existed, classical strains were last detected in southern Bangladesh in 1990^6^. However, factors associated with the replacement of the classical biotype as the predominant epidemic strain by the El Tor biotype, and eventual disappearance of the classical strains have not been adequately explained. The displacement of the classical strains by the El Tor might have been driven by undefined environmental forces to which the El Tor strains adapted more efficiently. For example, the propensity to form biofilms on aquatic surfaces, interactions with marine animals and plants, and the ability to resist acid production in carbohydrate metabolism are some of the possible determining factors^7^–^10^ in this ecological competition.

The recent recognition that phage predation may play a role in the natural control of cholera epidemics^2^,^11^,^12^ reinforces prediction that changes in this pathogen may also be driven by phages. The emergence of certain strains are likely to be enhanced by phages through the bactericidal mechanism in which phage sensitive strains are killed while providing a selective advantage to phage resistant strains. Therefore, the ability to evade phage predation constitutes important development in attaining evolutionary fitness. In the present study, we describe a bacteriophage which could have contributed to the range of factors causing the replacement of the 6^th^ pandemic classical strains of *V. cholerae* with the El Tor strains causing the current 7^th^ pandemic. We have also attempted to reproduce the conditions using microcosms and animal models, under which one or more phages might have acted in the events leading to the replacement of the classical biotype strains by the El Tor strains.

## Material and Methods

### 

#### Bacterial strains and phages:

*V. cholerae* strains used as indicators in plaque assays or host for phage preparations were from either clinical or environmental sources. Clinical strains were obtained from patients who attended the treatment center of the International Centre for Diarrhoeal Disease Research, Bangladesh (ICDDR, B) located in Dhaka or from the culture collection of ICDDR, B Environmental isolates were from surface waters in Dhaka. All *V. cholerae* strains were isolated between 1963 and 2009 and maintained in our collection.

*Isolation and estimation of phage*: A panel of *V. cholerae* O1 were used as potential indicator strains to detect and isolate classical biotype specific phage from environmental water using the plaque assay, as described previously^2^,^11^. Plaques were counted to estimate the concentration of phage particles in the sample. Phages from representative plaques were further purified, and the specificity of each phage was tested using a panel of strains belonging to different *Vibrio* species and serotypes. For detection and quantification of phage in intestinal extracts of mice, aliquots of the samples were pre-filtered through 0.22 μm pore size filters (Millipore Corporation, Bedford, MA) and dilutions of this filtrate were used in plaque assays. Animal studies were reviewed and approved by the Animal Experimental Ethics Committee of ICDDR, B.

#### Phage stability:

The effect of temperature, *p*H, and salinity on the stability of JSF9 phage was assessed by adding a defined number of phage particles into SM buffer (100 mM NaCl, 8 mM MgSO_4_, 50 mM Tris-Cl, *p*H 7.5) adjusted to different conditions. The titre of phage particles remaining after 6 h was expressed as a percentage of the original titre.

#### Electron microcopy of phage particles:

A high titre phage preparation [~10^10^ plaque forming unit/ml] was obtained using the plate lysis procedure as described previously^13^. The phage particle was negatively stained with 2 per cent uranyl acetate and was examined under a Philips transmission electron microscope (model 420T).

#### Determination of infectious dose (ID_50_) in mice:

To determine the ID_50_ of *V. cholerae* strains, 5-day-old Swiss Albino mice in groups of five were inoculated intragastrically with one or more laboratory grown *V. cholerae* strains, as well as with a mixture of *V. cholerae* and phage whenever appropriate. Aliquots of these samples were also cultured as described above to determine the cell count, and plaque assays were conducted to determine the concentration of phage. Mice were euthanized at 24 h and the small intestines were homogenized in phosphate buffered saline (PBS) *p*H 7.5. The homogenate was centrifuged and the pellet was re-suspended in fresh PBS, to eliminate possible phage particles (derived from phage positive samples) which could negatively influence the cell count. Dilutions of the suspension were then plated on taurocholate tellurite gelatine agar plates^14^ (70 μg/ml) to determine the number of colony forming units (cfu) of bacteria. The percentage of infected mice was plotted against the input dose to determine the ID_50_ as described previously^15^,^16^.

#### Competition assays in animals and in water samples:

To reproduce conditions under which phages might influence the selective enrichment or elimination of particular strains of pathogenic *V. cholerae*, we developed an assay system comprising repeated alternate inoculation of a mixture of *V. cholerae* strains and phage in animal models as well as in surface water samples. Competition assays were conducted by mixing 1:1 (v/v) ratio of the El Tor and classical biotype strains grown in Luria Bertini (LB) medium to log phase, and 10^6^ plug forming units (pfu) of the classical biotype specific phage JSF9. Aliquots (50 μl) of a 1,00-fold dilution of this mixture, were administered to 3-5-day-old Swiss Albino mice. A control preparation of the bacterial mixtures without the phage was also used in each batch of assay. Mice were euthanized at 24 h and the small intestines were homogenized in PBS. The homogenate was inoculated in filter-sterilized environmental water samples (1:100 dilution), and incubated at room temperature for 24 h. Aliquots (50 μl) of this water samples were inoculated into a second batch of mice, and the process was repeated. After each passage, the relative concentrations of the two strains in the intestinal contents of mice, as well as in the water samples were determined by plating dilutions of the samples on nutrient agar plates containing an appropriate antibiotic. The *V. cholerae* strains could be differentiated by their antibiotic resistance property since the El Tor strains used were resistant to nalidixic acid, and the classical strains used were resistant to tetracycline or streptomycin. For phage positive samples, the presence of phage and the possible emergence of phage resistant derivatives of the classical *V. cholerae* strains was also monitored as described previously^17^.

#### Transposon mutagenesis:

Transposon mutagenesis was conducted to create mutants of phage susceptible *V. cholerae* strains that became phage resistant. Mutation was done by mobilizing transposon pSC137 from the donor strain *E. coli* SM10λ_pir_ to the recipient strain by mating using previously described methods^18^. The mutants were screened for susceptibility to JSF9 phage to select mutants that became phage resistant.

## Results

### 

#### General properties of JSF9 phage:

The JSF9 phage was initially isolated from a sample of river water collected in Dhaka city, but are commonly found in different environmental water samples. The phage produced clear plaques with a diameter of approximately 1 mm on lawns of classical biotype *V. cholerae* O1 strains. When grown in LB with a classical biotype strain S224, the phage produced in the culture supernatant had a titre of ~10^8^ pfu/ml. The specificity of the phage was examined using a panel of bacterial strains belonging to different *Vibrio* species or serogroups. Strains belonging to the classical biotype of *V. cholerae* O1 (Table I) were susceptible to the phage, whereas all other strains tested were clearly resistant.

**Table I:** Susceptibility of different *V. cholerae* strains to infection by JSF9 phage

Strain	Description	Phage sensitivity*
S224, O395, S262, S263, L355, L396, L547, L362, B36921, C19385, C19751, D19316, E14850, E14983, AE4727, AE2883, AE4731, AE7471, AE7485	*V. cholerae* O1 classical biotype strains	S
C6706, 2924281, 2868921, 2868912, AF-1471, AL-30457, AM-33126, AN-32320, G-8747, G-3669, AP-13543, AM-33122, MG-116955, AN-24088, AO-29054, 2614, 3055, 112V214	*V. cholerae* O1 El Tor biotype strains	R
C-0273, C-1771, C-0270	*V. cholerae* O139 strains	R
V46, V51	*V. cholerae* O141 strains	R
SSF3 (S-224, VC395-0087::TnFC)	Derivative of classical biotype strain S224 with transposon insertion in gene encoding Glycerol 3-P-acyltransferase	R
SSF4 (O395, VC395-0087::TnFC)	Derivative of classical biotype strain O395 with transposon insertion in gene encoding Glycerol 3-P-acyltransferase	R
SSF6 (S-224, VC395-0291::TnFC)	Derivative of classical biotype strain S224 with transposon insertion in gene encoding UDP-glucose-4-epimerase	R

* Equal number of phage (~8.1 × 10^8^) were plated on different bacterial lawns. S, susceptible (plaque counts, 6.9 × 10^8^ – 8.1 × 10^8^); R, resistant (no plaques detected)

We further examined whether the resistance of different *V. cholerae* strains to JSF9 phage was due to possible lysogeny of the phage in these strains, causing the bacteria to become immune to further infection by JSF9 phage. All strains listed in Table I, were probed with JSF9 phage DNA in colony blot hybridization. However, none of the strains tested were found to carry the JSF9 phage genome (data not shown).

**Fig. 1:**
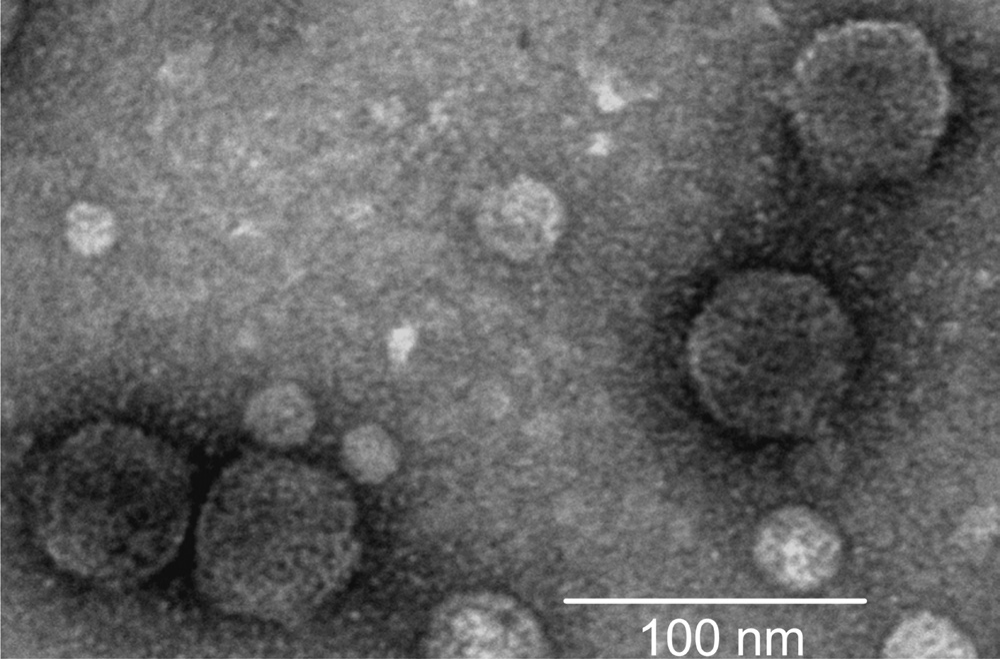
Electron micrograph of phage JSF9. The phage has a hexagonal head and a short tail, and belongs to the family *Podoviridae*.

Electron microscopic examination of JSF9 phage (Fig. 1) revealed that the phage particle comprised of a hexagonal head (~45 nm) and a short tail (~5 nm), and thus morphologically belonged to the family *Podoviridae*^19^. The genome of JSF9 phage consisted of a ~ 41 kb double stranded DNA. The phage was highly stable (75 to 95%) in a pH range between 3.0 and 9.0, and at temperatures below 37°C. At temperatures above 50°C, the phage particles were rapidly inactivated. JSF9 phage remained infectious for more than 10 wk when stored at room temperature in samples of filter-sterilized surface water samples collected in Dhaka. These findings suggested that JSF9 phage may persist in the aquatic environment as infectious particles and act on susceptible *V. cholerae* strains.

#### El Tor biotype strains out-compete classical strains in the presence of JSF9 phage:

We conducted competition assays between pairs of El Tor and classical biotype strains (Table II) in the presence and absence of JSF9 phage. Assays were designed to reproduce likely conditions in environmental water and host intestinal milieu (infant mouse model) which pathogenic *V. cholerae* strains are presumed to experience in nature. In the absence of JSF9 phage, classical strains competed well and the cumulative competition index (CCI) of El Tor to classical strain at the end of two consecutive rounds of assays in mice ranged between 1.15 and 1.84 (Table II), suggesting a somewhat better fitness of the El Tor strains. However, the relative fitness of El Tor strains was drastically enhanced when JSF9 phage was present, and the CCI ranged between 4.61 × 10^4^ and 3.13 × 10^5^ for different pairs of strains. Thus, the El Tor strains competed at least 10,000 fold better than classical strains in the presence of JSF9 phage.

**Table II:** Assays in microcosms and infant mice to model the effect of JSF9 phage on the emergence of the El Tor biotype and replacement of classical biotype of *V. cholerae* O1

Rounds of assays	El Tor strain 2680370		Classical strain O395		Phage JSF9		Cumulative competition index^c^
	Input count	Output count^a^	Input count	Output count^a^	Input count	Output count^a^	
Round 1A	9.5 × 10^5^	5.0 × 10^7^	1.2 × 10^6^	3.1 × 10^7^	0	0	1.84
Round 1B^b^	3.8 × 10^5^	1.2 × 10^7^	2.0 × 10^5^	8.2 × 10^6^	0	0	
Round 2A	9.2 × 10^5^	1.0 × 10^8^	1.1 × 10^6^	8.9 × 10^5^	7.8 × 10^7^	6.0 × 10^7^	8.85 × 10^4^
Round 2B b	6.0 × 10^5^	3.7 × 10^7^	2.0 × 10^3^	5.0 × 10^2^	7.8 × 10^5^	6.5 × 10^6^	

Rounds of assay	El Tor strain 2706062		Classical strain AE7471		Phage JSF9		Cumulative competition index^c^
	Input count	Output count^a^	Input count	Output count^a^	Input count	Output count^a^	

Round 3A	7.3 × 10^5^	2.2 × 10^7^	3.2 × 10^5^	2.6 × 10^6^	0	0	1.15
Round 3B^b^	4.0 × 10^4^	6.3 × 10^6^	8.0 × 10^4^	2.4 × 10^6^	0	0	
Round 4A	6.8 × 10^5^	3.4 × 10^7^	6.5 × 10^5^	4.8 × 10^5^	7.0 × 10^7^	7.5 × 10^7^	4.61 × 10^4^
Round 4B^b^	8.2 × 10^5^	2.6 × 10^7^	1.0 × 10^3^	5.4 × 10^2^	3.2 × 10^5^	1.0 × 10^6^	

Rounds of assay	El Tor strain 2680335		Classical strain AE2883		Phage JSF9		Cumulative competition index^c^
	Input count	Output count a	Input count	Output count a	Input count	Output count^a^	

Round 5A	6.0 × 10^5^	5.5 × 10^6^	5.6 × 10^5^	2.7 × 10^6^	0	0	1.73
Round 5B^b^	7.8 × 10^4^	4.1 × 10^6^	4.8 × 10^4^	2.2 × 10^6^	0	0	
Round 6A	6.0 × 10^5^	1.5 × 10^7^	6.8 × 10^5^	8.9 × 10^5^	9.0 × 10^7^	8.1 × 10^7^	3.13 × 10^5^
Round 6B^b^	8.0 × 10^4^	8.3 × 10^6^	7.0 × 10^3^	3.0 × 10	9.0 × 10^5^	5.0 × 10^5^	

a Concentration of *V. cholerae* cells and phage in the intestinal contents of mice challenged with a mixture of an El Tor and a classical strain with or without phage JSF9. Values represent the mean of 5 observations;

b bIntestinal contents of mice from round-A of the assay was inoculated into aliquots of filter-sterilized environmental water and incubated for 6 h. In round-B of the assay mice were inoculated with fractions of this water. All classical strains isolated from mice after both rounds remained sensitive to JSF9;

c For calculation of cumulative competition index for each set of assay the ratio of output counts of El Tor strain to classical strain in round-B was divided by the ratio of input counts of El Tor strain to classical strain in round-A

To analyze whether the increase in the competition index was due to a difference in the infectivity of the two biotypes in the presence of JSF9 phage, we determined the ID_50_ of the strains, a dose at which 50 per cent of challenged mice would be infected. This was done by inoculating various dilutions of log phase cultures into groups of mice with or without JSF9 phage. The mean ID_50_ of the two groups of strains were found to be similar in the absence of the phage. However, in the presence of JSF9 phage, the ID_50_ of the classical strains were ~10 fold higher (data not shown).

#### JSF9 phage resistant derivatives of classical strains are defective in colonization:

**Fig. 2:**
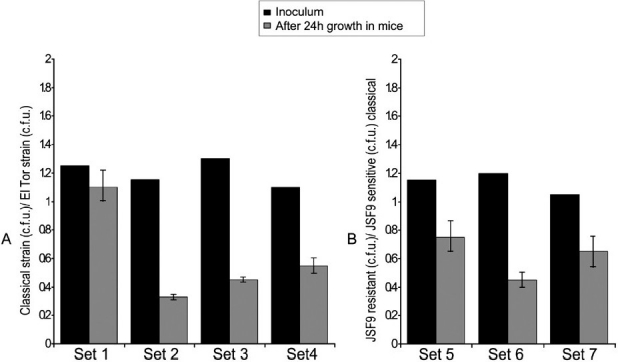
Competition assays in infant mice for colonization by different *V. cholerae* strains. **(A)** JSF9 phage resistant *V. cholerae* O1 El Tor strain C6706 and different derivatives of the classical strain S224 (see Table I); Set 1, S224 wild type; Set 2, spontaneous JSF phage resistant mutant; Set3, SSF3 (S-224, VC395-0087::TnFC); Set 4, SSF6 (S-224, VC395-0291::TnFC). **(B)** JSF9 phage sensitive classical strain S224 and different resistant derivatives of the same strain; Set 5, spontaneous JSF phage resistant mutant; Set 6, SSF3 (S-224, VC395-0087::TnFC); Set 7, SSF6 (S-224, VC395-0291::TnFC). Each mixture of strains was assayed in 5 different mice.

During the competition assays, spontaneous mutants of classical biotype strains resistant to JSF9 phage were detected. However, these mutants did not compete well with the El Tor biotype strains or the wild type classical strains in the mouse competition assay (Fig. 2). Additionally, transposon mutagenesis was conducted in two JSF9 phage susceptible classical *V. cholerae* strains S224 and O395, to obtain mutants that became phage resistant and the JSF9 phage did not form plaques on a lawn of the mutants. Chromosomal sequence analyses of the transposon insertion sites revealed that mutants of classical biotype strains which became resistant to JSF9 phage infection and were no longer able to adsorb phage particles carried insertions in phospholipid and lipopolysaccharide biosynthetic genes. The gene encoding glycerol 3-phosphate acyltransferase (GPAT) was most frequently hit (4 of 7 mutants characterized) among the phage resistant mutants, followed by the gene encoding UDP-glucose-4-epimerase (3 of 7 phage resistant mutants). These mutants showed similar defect in the mouse colonization assay (Fig. 2).

## Discussion

Genetic and ecological factors which led to the elimination of the classical biotype of *V. cholerae* and promoted the emergence of the El Tor biotype strains have not been adequately explained. The evolutionary fitness of pathogenic strains of *V. cholerae* are presumed to be enhanced by their ability to amplify in the human host^4^. However, both classical and El Tor biotypes of *V. cholerae* O1 produce the major virulence factors responsible for human colonization and dissemination through diarrhoeal purge. Hence the evolutionary success of the 7^th^ pandemic clone over the pre-existing 6^th^ pandemic strains is likely to have been influenced by factors other than their virulence potential in humans.

Recent microarray based comparative genomics have led to the identification of two clusters of genes in the 7^th^ pandemic El Tor strains, known as the Vibrio seventh pandemic islands (VSP-1 and VSP-2)^20^. Although these genes have been speculated to have a role in environmental fitness of El Tor strains, the precise functions of the genes remains to be investigated. The inherent capability of El Tor biotype strains to resist acid production in carbohydrate metabolism has also been suggested to be a factor improving their fitness^10^. Studies have demonstrated that phages can significantly contribute to the distribution and abundance of different clones of V. cholera^2^,^11^. The present study was designed to investigate whether JSF9 or similar phages might have contributed to the events that led to the replacement of the 6^th^ pandemic classical strains by the 7^th^ pandemic El Tor strains by a bactericidal selective process.

Pathogenic strains of *V. cholerae* are known to transit between two different habitats. These include the host intestinal milieu and the aquatic environment, and in both these habitats the bacteria encounter different adverse conditions. The assay system in which *V. cholerae* recovered from the intestines of mice pre-challenged with mixtures of the pathogens and the phage were inoculated into samples of environmental water, was designed to reproduce possible conditions under which the classical and El Tor strains might have competed for survival. Although in the absence of JSF9 phage, the classical biotype strains were able to compete with the El Tor biotype strains, the presence of JSF9 phage led the El Tor strains to out-compete the classical strains (Table II) suggesting clearly that JSF9 phage, in addition to possible other factors, could have markedly influenced the enrichment of El Tor strains and the elimination of classical strains.

Phage bacterial interactions appear to occur in both the aquatic environment and in the host intestine. Previous studies have shown that cholera victims excrete high titres of phage in their stools at a time that also correlates with the appearance of high concentrations of phage in nearby environmental waters^11^. Since pathogenic *V. cholerae* can grow rapidly in the host intestine, the host contributes significantly to the phage amplification by providing substrates for phage growth. Thus, host susceptibility to *V. cholerae* can influence phage-mediated selection of particular clones that are killed by the same phage once encountered in the extra-host environment^2^,^11^. However, if a bacterial clone (*e.g*., the El Tor 7^th^ pandemic clone) is resistant to a phage that otherwise kills a competing clone (*i.e*., the classical biotype clone), then the ecological fitness of the former would triumph over the latter in any dynamic setting that amplifies both bacteria and phage, while supporting the killing of phage-susceptible clones. Such an environment clearly exists in cholera endemic areas such as Bangladesh^2^,^11^. This strategy can result in elimination of the phage’s substrate organism and in the end the phage as well, unless the phage has a secondary host. Perhaps this logic also explains the absence of many classical biotype specific phages in the Bangladesh environment, at the same time suggesting that JSF9 phage might have an alternative host.

The bacterial surface structure often used by phages as a receptor for adsorption to bacteria includes lipopolysaccharide O-antigen, outer membrane proteins, and pili. Since both classical and El Tor biotype strains share the same O1 antigen and most other surface structures, the ability of the El Tor strains to survive attack by JSF9 phage under conditions when classical strains are completely susceptible indicates that the receptor for JSF9 phage is probably absent from the El Tor strains, and this might have been the key to the selective emergence of the El Tor strain. It has been recognized previously that there are certain differences in the pattern of gene expression between the El Tor and classical biotypes of *V. cholerae* O1^21^.

Although this study did not precisely identify the receptor for JSF9 phage infection, the observation that mutation in genes for glycerol 3-phosphate acyltransferase and UDP-glucose-4-epimerase confer phage resistance indicates a possible role of the bacterial LPS in JSF9 phage susceptibility. Alternatively, an OMP whose assembly in the membrane depends on lipid synthesis may also have a role. For example, in an *Escherichia coli* strain which is defective in glycerol-3-phosphate acyltransferase, a marked decrease of insertion of the bacteriophage Lambda receptor LamB protein occurs into the outer membrane^22^.

In conclusion, the results of this study suggest that the ability to resist predation by JSF9 or similar phages might have contributed to the evolutionary fitness of the *V. cholerae* El Tor biotype compared to that of the classical biotype both as a global cause of cholera and as an environmental organism.
